# Supplementary material for: Modeling glioblastoma heterogeneity as a dynamic network of cell states
Source: Mol Syst Biol. 2021 Sep 16;17(9):e10105. doi: 10.15252/msb.202010105 (PMC8444284; doi:10.15252/msb.202010105)
Supplement: Supplementary file 6 — Source Data for Figure 5 [file MSB-17-e10105-s004.zip › Figure5A_sourcedata/GSEA_3071/nosigdb_stateI.GseaPreranked.1622795145664/gsea_report_for_na_neg_1622795145664.html]

Report for na\_neg 1622795145664 [GSEA]

| GS  follow link to MSigDB | GS DETAILS | SIZE | ES | NES | NOM p-val | FDR q-val | FWER p-val | RANK AT MAX | LEADING EDGE || 1 | LARSSON\_STATE\_4 | Details ... | 96 | -0.73 | -3.80 | 0.000 | 0.000 | 0.000 | 831 | tags=90%, list=24%, signal=114% |
| 2 | RICHARDS\_NOWAKOWSKI\_SCIENCE\_2017\_MGE\_IPC2\_UPREG | Details ... | 352 | -0.58 | -3.65 | 0.000 | 0.000 | 0.000 | 1149 | tags=76%, list=33%, signal=101% |
| 3 | DOROSZKO\_U3180MG\_CLUSTER\_3 | Details ... | 349 | -0.57 | -3.56 | 0.000 | 0.000 | 0.000 | 1001 | tags=68%, list=29%, signal=86% |
| 4 | FELDMAN\_2020\_S-G2 | Details ... | 265 | -0.59 | -3.55 | 0.000 | 0.000 | 0.000 | 941 | tags=71%, list=27%, signal=90% |
| 5 | DOROSZKO\_U3013MG\_CLUSTER\_2 | Details ... | 169 | -0.62 | -3.52 | 0.000 | 0.000 | 0.000 | 878 | tags=74%, list=25%, signal=94% |
| 6 | IVY\_CT | Details ... | 117 | -0.66 | -3.52 | 0.000 | 0.000 | 0.000 | 507 | tags=60%, list=14%, signal=68% |
| 7 | DOROSZKO\_U3054MG\_CLUSTER\_4 | Details ... | 410 | -0.56 | -3.52 | 0.000 | 0.000 | 0.000 | 1181 | tags=73%, list=34%, signal=97% |
| 8 | RICHARDS\_NOWAKOWSKI\_SCIENCE\_2017\_MGE\_DIV\_UPREG | Details ... | 417 | -0.54 | -3.41 | 0.000 | 0.000 | 0.000 | 1181 | tags=71%, list=34%, signal=95% |
| 9 | GAROFANO\_IAVARONE\_PPR\_SUBTYPE | Details ... | 269 | -0.54 | -3.27 | 0.000 | 0.000 | 0.000 | 1328 | tags=78%, list=38%, signal=116% |
| 10 | FELDMAN\_2020\_G1 | Details ... | 138 | -0.59 | -3.26 | 0.000 | 0.000 | 0.000 | 921 | tags=76%, list=26%, signal=99% |
| 11 | FELDMAN\_2020\_S | Details ... | 123 | -0.59 | -3.21 | 0.000 | 0.000 | 0.000 | 957 | tags=76%, list=27%, signal=101% |
| 12 | SINGH\_SOX2\_UPREGULATED\_TRANSCRIPTS\_PMID\_28122245 | Details ... | 186 | -0.55 | -3.20 | 0.000 | 0.000 | 0.000 | 473 | tags=42%, list=13%, signal=46% |
| 13 | VANGRONINGEN\_ADRN | Details ... | 174 | -0.56 | -3.19 | 0.000 | 0.000 | 0.000 | 699 | tags=56%, list=20%, signal=66% |
| 14 | RICHARDS\_ZHONG\_NATURE\_2018\_NPCS\_UPREG | Details ... | 113 | -0.58 | -3.12 | 0.000 | 0.000 | 0.000 | 846 | tags=69%, list=24%, signal=88% |
| 15 | NB\_KI\_CLUSTER5 | Details ... | 80 | -0.63 | -3.11 | 0.000 | 0.000 | 0.000 | 728 | tags=68%, list=21%, signal=83% |
| 16 | RICHARDS\_VERHAAK\_CANCERCELL\_2010\_PRONEURAL | Details ... | 70 | -0.63 | -3.09 | 0.000 | 0.000 | 0.000 | 482 | tags=50%, list=14%, signal=57% |
| 17 | SEURAT\_CELLCYCLE | Details ... | 83 | -0.61 | -3.05 | 0.000 | 0.000 | 0.000 | 1181 | tags=86%, list=34%, signal=126% |
| 18 | RICHARDS\_NOWAKOWSKI\_SCIENCE\_2017\_IPC\_DIV1\_UPREG | Details ... | 415 | -0.48 | -3.00 | 0.000 | 0.000 | 0.000 | 1159 | tags=62%, list=33%, signal=82% |
| 19 | SUVA\_NPC1\_PMID\_31327527 | Details ... | 28 | -0.74 | -2.92 | 0.000 | 0.000 | 0.000 | 458 | tags=64%, list=13%, signal=73% |
| 20 | GAROFANO\_IAVARONE\_LIST\_OF\_NEURAL\_PROGENITOR\_MARKER\_GENES | Details ... | 77 | -0.59 | -2.92 | 0.000 | 0.000 | 0.000 | 732 | tags=58%, list=21%, signal=72% |
| 21 | RICHARDS\_NOWAKOWSKI\_SCIENCE\_2017\_MGE\_RG2\_UPREG |  | 257 | -0.49 | -2.92 | 0.000 | 0.000 | 0.000 | 921 | tags=58%, list=26%, signal=72% |
| 22 | RICHARDS\_NOWAKOWSKI\_SCIENCE\_2017\_MGE\_IPC3\_UPREG |  | 76 | -0.59 | -2.90 | 0.000 | 0.000 | 0.000 | 752 | tags=59%, list=21%, signal=74% |
| 23 | FELDMAN\_2020\_G2-M |  | 214 | -0.49 | -2.87 | 0.000 | 0.000 | 0.000 | 994 | tags=64%, list=28%, signal=83% |
| 24 | OLSEN\_ADRENERGIC |  | 52 | -0.63 | -2.82 | 0.000 | 0.000 | 0.000 | 451 | tags=48%, list=13%, signal=54% |
| 25 | RICHARDS\_NEFTEL\_CELL\_2019\_NPC1 |  | 28 | -0.74 | -2.82 | 0.000 | 0.000 | 0.000 | 458 | tags=64%, list=13%, signal=73% |
| 26 | RICHARDS\_NEFTEL\_CELL\_2019\_G1\_S |  | 26 | -0.73 | -2.80 | 0.000 | 0.000 | 0.000 | 457 | tags=73%, list=13%, signal=83% |
| 27 | FELDMAN\_2020\_NEURAL G0 |  | 254 | -0.46 | -2.78 | 0.000 | 0.000 | 0.000 | 819 | tags=52%, list=23%, signal=63% |
| 28 | RICHARDS\_NOWAKOWSKI\_SCIENCE\_2017\_MGE\_IPC1\_UPREG |  | 328 | -0.45 | -2.77 | 0.000 | 0.000 | 0.000 | 1225 | tags=64%, list=35%, signal=90% |
| 29 | RICHARDS\_NOWAKOWSKI\_SCIENCE\_2017\_NIN1\_UPREG |  | 89 | -0.55 | -2.75 | 0.000 | 0.000 | 0.000 | 615 | tags=52%, list=18%, signal=61% |
| 30 | SUVA\_G1S\_PMID\_31327527 |  | 26 | -0.73 | -2.72 | 0.000 | 0.000 | 0.000 | 457 | tags=73%, list=13%, signal=83% |
| 31 | SUVA\_NPC2\_PMID\_31327527 |  | 21 | -0.73 | -2.62 | 0.000 | 0.000 | 0.000 | 436 | tags=71%, list=12%, signal=81% |
| 32 | FELDMAN\_2020\_M-EARLYG1 |  | 86 | -0.52 | -2.62 | 0.000 | 0.000 | 0.000 | 752 | tags=52%, list=21%, signal=65% |
| 33 | RICHARDS\_NEFTEL\_CELL\_2019\_NPC2 |  | 21 | -0.73 | -2.58 | 0.000 | 0.000 | 0.000 | 436 | tags=71%, list=12%, signal=81% |
| 34 | RICHARDS\_NOWAKOWSKI\_SCIENCE\_2017\_IN\_CTX\_CGE2\_UPREG |  | 107 | -0.49 | -2.53 | 0.000 | 0.000 | 0.000 | 629 | tags=45%, list=18%, signal=53% |
| 35 | RICHARDS\_ZHONG\_NATURE\_2018\_OPC\_UPREG |  | 49 | -0.55 | -2.49 | 0.000 | 0.000 | 0.000 | 350 | tags=37%, list=10%, signal=40% |
| 36 | RICHARDS\_NOWAKOWSKI\_SCIENCE\_2017\_ASTROCYTE\_UPREG |  | 259 | -0.42 | -2.49 | 0.000 | 0.000 | 0.000 | 358 | tags=27%, list=10%, signal=27% |
| 37 | DALMO\_BARCODENUMBER\_UNPUB |  | 137 | -0.45 | -2.47 | 0.000 | 0.000 | 0.000 | 752 | tags=52%, list=21%, signal=63% |
| 38 | LARSSON\_STATE\_6 |  | 51 | -0.56 | -2.46 | 0.000 | 0.000 | 0.000 | 287 | tags=35%, list=8%, signal=38% |
| 39 | ALMSTEDT\_RISK |  | 24 | -0.67 | -2.45 | 0.000 | 0.000 | 0.000 | 383 | tags=50%, list=11%, signal=56% |
| 40 | RICHARDS\_NOWAKOWSKI\_SCIENCE\_2017\_IN\_CTX\_CGE1\_UPREG |  | 108 | -0.47 | -2.45 | 0.000 | 0.000 | 0.000 | 977 | tags=54%, list=28%, signal=72% |
| 41 | RICHARDS\_NOWAKOWSKI\_SCIENCE\_2017\_NIN4\_UPREG |  | 58 | -0.51 | -2.41 | 0.000 | 0.000 | 0.000 | 302 | tags=29%, list=9%, signal=32% |
| 42 | RICHARDS\_NOWAKOWSKI\_SCIENCE\_2017\_NIN3\_UPREG |  | 73 | -0.50 | -2.40 | 0.000 | 0.000 | 0.000 | 431 | tags=36%, list=12%, signal=40% |
| 43 | POLLEN\_OUTERRADIALGLIA\_PMID\_26406371 |  | 33 | -0.60 | -2.38 | 0.000 | 0.000 | 0.000 | 379 | tags=42%, list=11%, signal=47% |
| 44 | OUTERRADIALGLIA |  | 33 | -0.60 | -2.38 | 0.000 | 0.000 | 0.000 | 379 | tags=42%, list=11%, signal=47% |
| 45 | RICHARDS\_NOWAKOWSKI\_SCIENCE\_2017\_IN\_CTX\_MGE1\_UPREG |  | 123 | -0.44 | -2.36 | 0.000 | 0.000 | 0.000 | 782 | tags=44%, list=22%, signal=55% |
| 46 | TISIG\_PMID\_32649888 |  | 23 | -0.65 | -2.36 | 0.000 | 0.000 | 0.000 | 352 | tags=52%, list=10%, signal=58% |
| 47 | RICHARDS\_NEFTEL\_CELL\_2019\_AC |  | 24 | -0.63 | -2.34 | 0.000 | 0.000 | 0.000 | 358 | tags=42%, list=10%, signal=46% |
| 48 | RICHARDS\_NEFTEL\_CELL\_2019\_G2\_M |  | 38 | -0.55 | -2.33 | 0.000 | 0.000 | 0.000 | 1312 | tags=87%, list=37%, signal=137% |
| 49 | ALLEN\_112\_ASTROL1FGFR3MT1G |  | 17 | -0.69 | -2.32 | 0.000 | 0.000 | 0.000 | 348 | tags=59%, list=10%, signal=65% |
| 50 | SUVA\_AC\_PMID\_31327527 |  | 24 | -0.63 | -2.31 | 0.000 | 0.000 | 0.000 | 358 | tags=42%, list=10%, signal=46% |
| 51 | SUVA\_G2M\_PMID\_31327527 |  | 38 | -0.55 | -2.30 | 0.000 | 0.000 | 0.000 | 1312 | tags=87%, list=37%, signal=137% |
| 52 | RICHARDS\_NOWAKOWSKI\_SCIENCE\_2017\_IPC\_NEN1\_UPREG\_1 |  | 61 | -0.49 | -2.30 | 0.000 | 0.000 | 0.000 | 737 | tags=46%, list=21%, signal=57% |
| 53 | RICHARDS\_NOWAKOWSKI\_SCIENCE\_2017\_OPC\_UPREG |  | 182 | -0.40 | -2.27 | 0.000 | 0.000 | 0.001 | 535 | tags=31%, list=15%, signal=34% |
| 54 | RICHARDS\_NOWAKOWSKI\_SCIENCE\_2017\_RG\_EARLY\_UPREG |  | 249 | -0.38 | -2.27 | 0.000 | 0.000 | 0.001 | 941 | tags=47%, list=27%, signal=60% |
| 55 | RICHARDS\_NOWAKOWSKI\_SCIENCE\_2017\_VRG\_UPREG |  | 215 | -0.38 | -2.26 | 0.000 | 0.000 | 0.001 | 413 | tags=27%, list=12%, signal=28% |
| 56 | RICHARDS\_NEFTEL\_CELL\_2019\_OPC |  | 22 | -0.62 | -2.25 | 0.000 | 0.000 | 0.001 | 84 | tags=32%, list=2%, signal=32% |
| 57 | SUVA\_OPC\_PMID\_31327527 |  | 22 | -0.62 | -2.24 | 0.000 | 0.000 | 0.002 | 84 | tags=32%, list=2%, signal=32% |
| 58 | DOROSZKO\_U3054MG\_CLUSTER\_0 |  | 52 | -0.49 | -2.24 | 0.000 | 0.000 | 0.002 | 519 | tags=38%, list=15%, signal=44% |
| 59 | NB\_KI\_CLUSTER4 |  | 66 | -0.47 | -2.24 | 0.000 | 0.000 | 0.002 | 451 | tags=33%, list=13%, signal=38% |
| 60 | RICHARDS\_NOWAKOWSKI\_SCIENCE\_2017\_IPC\_NEN1\_UPREG |  | 119 | -0.41 | -2.20 | 0.000 | 0.000 | 0.005 | 553 | tags=35%, list=16%, signal=40% |
| 61 | RICHARDS\_ZHONG\_NATURE\_2018\_ASTROCYTES\_UPREG |  | 147 | -0.39 | -2.17 | 0.000 | 0.000 | 0.007 | 378 | tags=27%, list=11%, signal=29% |
| 62 | RICHARDS\_NOWAKOWSKI\_SCIENCE\_2017\_NEN\_EARLY2\_UPREG |  | 177 | -0.38 | -2.16 | 0.000 | 0.000 | 0.009 | 520 | tags=31%, list=15%, signal=34% |
| 63 | FELDMAN\_2020\_LATEG1 |  | 65 | -0.45 | -2.14 | 0.000 | 0.000 | 0.012 | 692 | tags=48%, list=20%, signal=58% |
| 64 | PARADA\_T2\_UP\_26461091 |  | 44 | -0.48 | -2.13 | 0.000 | 0.000 | 0.013 | 430 | tags=39%, list=12%, signal=43% |
| 65 | DOROSZKO\_U3013MG\_CLUSTER\_0 |  | 23 | -0.58 | -2.10 | 0.000 | 0.000 | 0.019 | 277 | tags=43%, list=8%, signal=47% |
| 66 | RICHARDS\_NOWAKOWSKI\_SCIENCE\_2017\_IN\_STR\_UPREG |  | 69 | -0.43 | -2.08 | 0.000 | 0.000 | 0.024 | 870 | tags=46%, list=25%, signal=60% |
| 67 | RICHARDS\_NOWAKOWSKI\_SCIENCE\_2017\_NIN5\_UPREG |  | 117 | -0.38 | -2.06 | 0.000 | 0.001 | 0.036 | 615 | tags=33%, list=18%, signal=39% |
| 68 | ALLEN\_113\_ASTROL1FGFR3FOS |  | 16 | -0.61 | -2.05 | 0.000 | 0.001 | 0.038 | 348 | tags=56%, list=10%, signal=62% |
| 69 | GAROFANO\_IAVARONE\_NEU\_SUBTYPE |  | 83 | -0.41 | -2.05 | 0.000 | 0.001 | 0.039 | 553 | tags=34%, list=16%, signal=39% |
| 70 | RICHARDS\_NOWAKOWSKI\_SCIENCE\_2017\_EN\_V1\_1\_UPREG |  | 313 | -0.33 | -2.03 | 0.000 | 0.001 | 0.046 | 643 | tags=29%, list=18%, signal=32% |
| 71 | RICHARDS\_CAHOY\_JNEUROSCI\_2010\_ASTRO\_YOUNG |  | 396 | -0.32 | -2.01 | 0.000 | 0.001 | 0.057 | 834 | tags=41%, list=24%, signal=48% |
| 72 | DOROSZKO\_PERIVASCULAR\_INVASION |  | 27 | -0.53 | -2.00 | 0.002 | 0.001 | 0.065 | 293 | tags=41%, list=8%, signal=44% |
| 73 | RICHARDS\_NOWAKOWSKI\_SCIENCE\_2017\_EN\_PFC1\_UPREG |  | 323 | -0.32 | -1.99 | 0.000 | 0.001 | 0.071 | 649 | tags=28%, list=18%, signal=32% |
| 74 | RICHARDS\_NOWAKOWSKI\_SCIENCE\_2017\_NEN\_EARLY1\_UPREG |  | 249 | -0.33 | -1.99 | 0.000 | 0.001 | 0.073 | 556 | tags=28%, list=16%, signal=31% |
| 75 | IVY\_IT |  | 29 | -0.50 | -1.98 | 0.002 | 0.001 | 0.082 | 302 | tags=34%, list=9%, signal=37% |
| 76 | RICHARDS\_NOWAKOWSKI\_SCIENCE\_2017\_EN\_PFC3\_UPREG |  | 127 | -0.36 | -1.96 | 0.000 | 0.001 | 0.100 | 497 | tags=29%, list=14%, signal=33% |
| 77 | RICHARDS\_NOWAKOWSKI\_SCIENCE\_2017\_EN\_V1\_2\_UPREG |  | 278 | -0.32 | -1.95 | 0.000 | 0.002 | 0.110 | 632 | tags=29%, list=18%, signal=33% |
| 78 | RICHARDS\_NOWAKOWSKI\_SCIENCE\_2017\_NIN2\_UPREG |  | 173 | -0.34 | -1.93 | 0.000 | 0.002 | 0.123 | 1213 | tags=50%, list=35%, signal=73% |
| 79 | TIISIG\_PMID\_32649888 |  | 22 | -0.53 | -1.93 | 0.003 | 0.002 | 0.126 | 255 | tags=36%, list=7%, signal=39% |
| 80 | RICHARDS\_NOWAKOWSKI\_SCIENCE\_2017\_IN\_CTX\_MGE2\_UPREG |  | 97 | -0.37 | -1.92 | 0.000 | 0.002 | 0.139 | 687 | tags=37%, list=20%, signal=45% |
| 81 | RICHARDS\_NOWAKOWSKI\_SCIENCE\_2017\_IPC\_NEN3\_UPREG |  | 111 | -0.36 | -1.90 | 0.001 | 0.002 | 0.169 | 613 | tags=32%, list=17%, signal=38% |
| 82 | RICHARDS\_NOWAKOWSKI\_SCIENCE\_2017\_EN\_PFC2\_UPREG |  | 336 | -0.30 | -1.83 | 0.000 | 0.004 | 0.272 | 844 | tags=35%, list=24%, signal=41% |
| 83 | FELDMAN\_2020\_LOWRNA |  | 489 | -0.28 | -1.83 | 0.000 | 0.005 | 0.298 | 941 | tags=37%, list=27%, signal=44% |
| 84 | TF\_CENSUS\_TAIPALE |  | 307 | -0.29 | -1.81 | 0.000 | 0.005 | 0.332 | 687 | tags=27%, list=20%, signal=31% |
| 85 | GANDAL\_ALCOHOLADDICTION\_DN\_PMID29439242 |  | 39 | -0.43 | -1.80 | 0.011 | 0.005 | 0.342 | 524 | tags=31%, list=15%, signal=36% |
| 86 | NB\_KI\_CLUSTER3 |  | 74 | -0.37 | -1.80 | 0.004 | 0.005 | 0.346 | 1788 | tags=84%, list=51%, signal=167% |
| 87 | WENG\_STEMNESS\_PMID\_30982771 |  | 20 | -0.50 | -1.79 | 0.012 | 0.006 | 0.382 | 436 | tags=40%, list=12%, signal=45% |
| 88 | LARSSON\_STATE\_2 |  | 54 | -0.39 | -1.76 | 0.005 | 0.008 | 0.456 | 316 | tags=22%, list=9%, signal=24% |
| 89 | RICHARDS\_NOWAKOWSKI\_SCIENCE\_2017\_NEN\_LATE\_UPREG |  | 118 | -0.33 | -1.75 | 0.001 | 0.008 | 0.466 | 767 | tags=34%, list=22%, signal=42% |
| 90 | ALLEN\_111\_ASTROL1\_6FGFR3ETNPPL |  | 15 | -0.54 | -1.74 | 0.012 | 0.009 | 0.513 | 317 | tags=47%, list=9%, signal=51% |
| 91 | IVY\_LE |  | 23 | -0.46 | -1.73 | 0.018 | 0.009 | 0.537 | 532 | tags=43%, list=15%, signal=51% |
| 92 | KLING\_GBM\_PHENO\_PMID\_27667176 |  | 84 | -0.34 | -1.68 | 0.015 | 0.014 | 0.699 | 397 | tags=27%, list=11%, signal=30% |
| 93 | DOROSZKO\_U3054MG\_CLUSTER\_6 |  | 62 | -0.35 | -1.66 | 0.009 | 0.016 | 0.747 | 1417 | tags=69%, list=40%, signal=114% |
| 94 | RICHARDS\_VERHAAK\_CANCERCELL\_2010\_CLASSICAL |  | 55 | -0.37 | -1.65 | 0.020 | 0.017 | 0.777 | 692 | tags=38%, list=20%, signal=47% |
| 95 | GANDAL\_BIPOLAR\_DN\_PMID29439242 |  | 15 | -0.51 | -1.65 | 0.043 | 0.017 | 0.781 | 383 | tags=40%, list=11%, signal=45% |
| 96 | RICHARDS\_NOWAKOWSKI\_SCIENCE\_2017\_EN\_V1\_3\_UPREG |  | 301 | -0.27 | -1.64 | 0.001 | 0.018 | 0.787 | 788 | tags=31%, list=22%, signal=36% |
| 97 | DOROSZKO\_U3054MG\_CLUSTER\_7 |  | 17 | -0.49 | -1.64 | 0.029 | 0.017 | 0.788 | 297 | tags=41%, list=8%, signal=45% |
| 98 | DOROSZKO\_U3054MG\_CLUSTER\_5 |  | 49 | -0.36 | -1.60 | 0.026 | 0.024 | 0.892 | 604 | tags=29%, list=17%, signal=34% |
| 99 | DOROSZKO\_U3180MG\_CLUSTER\_5 |  | 20 | -0.45 | -1.60 | 0.038 | 0.024 | 0.896 | 326 | tags=35%, list=9%, signal=38% |
| 100 | ALMSTEDT\_11Q\_MSIGDB |  | 106 | -0.30 | -1.56 | 0.013 | 0.030 | 0.957 | 510 | tags=25%, list=15%, signal=28% |
| 101 | RICHARDS\_NOWAKOWSKI\_SCIENCE\_2017\_TRG\_UPREG |  | 484 | -0.24 | -1.54 | 0.001 | 0.033 | 0.971 | 402 | tags=16%, list=11%, signal=16% |
| 102 | DOROSZKO\_U3180MG\_CLUSTER\_2 |  | 186 | -0.26 | -1.49 | 0.013 | 0.047 | 0.992 | 320 | tags=16%, list=9%, signal=17% |
| 103 | DOROSZKO\_U3180MG\_CLUSTER\_4 |  | 79 | -0.30 | -1.48 | 0.042 | 0.052 | 0.995 | 1445 | tags=63%, list=41%, signal=105% |
| 104 | RICHARDS\_NOWAKOWSKI\_SCIENCE\_2017\_MGE\_RG1\_UPREG |  | 171 | -0.26 | -1.45 | 0.026 | 0.060 | 0.997 | 413 | tags=19%, list=12%, signal=21% |
| 105 | GAROFANO\_IAVARONE\_MTC\_SUBTYPE |  | 73 | -0.30 | -1.43 | 0.053 | 0.067 | 0.998 | 632 | tags=29%, list=18%, signal=34% |
| 106 | RICHARDS\_CAHOY\_JNEUROSCI\_2009\_NEURON |  | 352 | -0.22 | -1.39 | 0.022 | 0.084 | 1.000 | 632 | tags=23%, list=18%, signal=26% |
| 107 | LARSSON\_STATE\_3 |  | 56 | -0.31 | -1.39 | 0.099 | 0.085 | 1.000 | 407 | tags=20%, list=12%, signal=22% |
| 108 | LARSSON\_STATE\_1 |  | 56 | -0.30 | -1.38 | 0.061 | 0.090 | 1.000 | 652 | tags=39%, list=19%, signal=47% |
| 109 | GANDAL\_BIPOLAR\_UP\_PMID29439242 |  | 40 | -0.32 | -1.35 | 0.108 | 0.103 | 1.000 | 720 | tags=38%, list=21%, signal=47% |
| 110 | RICHARDS\_LIDDELOW\_NATURE\_2017\_A2\_REACTIVE\_ASTROCYTES |  | 21 | -0.37 | -1.33 | 0.160 | 0.114 | 1.000 | 214 | tags=24%, list=6%, signal=25% |
| 111 | NB\_KI\_CLUSTER2 |  | 87 | -0.26 | -1.31 | 0.113 | 0.132 | 1.000 | 519 | tags=23%, list=15%, signal=26% |
| 112 | DOROSZKO\_U3013MG\_CLUSTER\_3 |  | 59 | -0.28 | -1.29 | 0.125 | 0.142 | 1.000 | 139 | tags=12%, list=4%, signal=12% |
| 113 | ALMSTEDT\_ALK |  | 35 | -0.31 | -1.26 | 0.194 | 0.163 | 1.000 | 608 | tags=31%, list=17%, signal=38% |
| 114 | GANDAL\_SCHIZOPHRENIA\_UP\_PMID29439242 |  | 54 | -0.27 | -1.26 | 0.157 | 0.163 | 1.000 | 262 | tags=19%, list=7%, signal=20% |
| 115 | RICHARDS\_CAHOY\_JNEUROSCI\_2011\_ASTRO\_MATURE |  | 212 | -0.21 | -1.24 | 0.111 | 0.181 | 1.000 | 339 | tags=13%, list=10%, signal=14% |
| 116 | DOROSZKO\_U3054MG\_CLUSTER\_10 |  | 121 | -0.22 | -1.21 | 0.179 | 0.205 | 1.000 | 441 | tags=18%, list=13%, signal=20% |
| 117 | RICHARDS\_CAHOY\_JNEUROSCI\_2008\_OLIGODENDROCYTE |  | 447 | -0.19 | -1.18 | 0.125 | 0.235 | 1.000 | 492 | tags=17%, list=14%, signal=18% |
| 118 | PARADA\_T1\_UP\_26461091 |  | 54 | -0.26 | -1.18 | 0.246 | 0.238 | 1.000 | 572 | tags=30%, list=16%, signal=35% |
| 119 | LARSSON\_STATE\_5 |  | 46 | -0.24 | -1.05 | 0.393 | 0.419 | 1.000 | 521 | tags=20%, list=15%, signal=23% |
| 120 | PARADA\_T2\_DN\_26461091 |  | 24 | -0.23 | -0.86 | 0.656 | 0.750 | 1.000 | 469 | tags=21%, list=13%, signal=24% |
| 121 | RICHARDS\_NOWAKOWSKI\_SCIENCE\_2017\_U3\_UPREG |  | 288 | -0.14 | -0.82 | 0.830 | 0.802 | 1.000 | 731 | tags=21%, list=21%, signal=24% |
| 122 | PARADA\_T1\_DN\_26461091 |  | 26 | -0.22 | -0.82 | 0.710 | 0.803 | 1.000 | 1051 | tags=46%, list=30%, signal=65% |
| 123 | WANG\_CLASSICAL\_PMID\_28697342 |  | 19 | -0.23 | -0.81 | 0.698 | 0.798 | 1.000 | 352 | tags=21%, list=10%, signal=23% |
| 124 | NB\_KI\_CLUSTER24 |  | 37 | -0.20 | -0.81 | 0.720 | 0.795 | 1.000 | 705 | tags=22%, list=20%, signal=27% |
| 125 | DOROSZKO\_U3054MG\_CLUSTER\_8 |  | 181 | -0.13 | -0.72 | 0.930 | 0.903 | 1.000 | 153 | tags=6%, list=4%, signal=6% |
| 126 | NB\_KI\_CLUSTER22 |  | 56 | -0.15 | -0.69 | 0.872 | 0.920 | 1.000 | 221 | tags=9%, list=6%, signal=9% |
| 127 | NB\_KI\_CLUSTER6 |  | 110 | -0.11 | -0.60 | 0.974 | 0.969 | 1.000 | 1408 | tags=43%, list=40%, signal=69% |
| 128 | NB\_KI\_CLUSTER7 |  | 49 | -0.09 | -0.39 | 0.995 | 0.998 | 1.000 | 1433 | tags=43%, list=41%, signal=71% |
Table: Gene sets enriched in phenotype **na **[plain text format]****
